# Amitraz and Its Metabolites: Oxidative Stress-Mediated Cytotoxicity in HepG2 Cells and Study of Their Stability and Characterization in Honey

**DOI:** 10.3390/antiox12040885

**Published:** 2023-04-05

**Authors:** Marialuce Giorgini, Mercedes Taroncher, Josefa Tolosa, María-José Ruiz, Yelko Rodríguez-Carrasco

**Affiliations:** Laboratory of Food Chemistry and Toxicology, Faculty of Pharmacy, University of Valencia, Av. Vicent Andrés Estellés s/n, 46100 Burjassot, Valencia, Spain

**Keywords:** amitraz, metabolites, cytotoxicity, oxidative stress, in vitro, 5-Hydroxymethylfurfural, honey, HPLC-QTOF

## Abstract

The population decrease of bees that has been observed in recent years due to the *Varroa destructor* parasite may endanger the production of bee-products whose demand is on the rise. To minimize the negative effects caused by this parasite, the pesticide amitraz is commonly used by beekeepers. Based on these, the objectives of this work are to determine the toxic effects caused by amitraz and its metabolites in HepG2 cells, as well as its determination in honey samples and the study of its stability with different heat treatments commonly used in the honey industry and its relationship with the amount of 5-hydroxymethylfurfural (HMF) produced. Amitraz significantly decreased cell viability by MTT assay and total protein content (PC) assay, being more cytotoxic than its metabolites. Amitraz and its metabolites caused oxidative stress by Lipid Peroxidation (LPO) production and Reactive Oxygen Species (ROS) generation. Residues of amitraz and/or its metabolites were found in analyzed honey samples, with 2,4-Dimethylaniline (2,4-DMA) being the main metabolite confirmed by high-performance liquid chromatography-high resolution mass spectrometry (HPLC-QTOF HRMS). Amitraz and its metabolites resulted as unstable even at moderate heat treatments. Additionally, a positive correlation in terms of HMF concentration in samples and the severity of heat treatment was also observed. However, quantified amitraz and HMF were within the levels set in the regulation.

## 1. Introduction

Honey bees (*Apis mellifera*) are the primary breaded pollinators for 75% of the food-producing crops worldwide, according to literature estimations [1]. The term bee-products refers to the end products made by honeybees. Bee-products have gained popularity because of their therapeutical and nutritional benefits [2]. At the global level, the most produced and consumed bee-product is honey. According to the latest data published by the Food and Agricultural Organization of the United Nations, a total of 1773 thousand tons of honey were produced in 2020 globally. With regard to consumption, the data indicate a honey per capita consumption of 0.2 kg; whereas a consumption three times higher than the global average is reported for Europeans [3]. Therefore, there are social, economic, and scientific worries over the global fall in bee populations [4]. The decline in honeybee numbers is caused by pesticides, illnesses, and mites that impact the colonies. These elements work together to induce colony collapse disorder, which is the primary reason for colony losses globally [5]. 

The parasite mite *Varroa destructor* has the most severe negative impact on honeybees of all the above-mentioned variables. In honeybee colonies that receive no treatment or inadequate treatment, heavy *Varroa* infection could result in massive deaths in the colonies within a few weeks. To manage varroosis, many acaricidal substances such as fluvalinate, acrinathrin, and amitraz are often used [6]. Among these acaricides, amitraz is the substance with the highest maximum residue level in honey (0.2 mg/kg), which is why it is usually the pesticide of choice for beekeepers [7]. 

Amitraz belongs to the class of formamidine pesticides. Due to the low pH of the hive and/or heat treatment of the honey, amitraz quickly undergoes biotransformation. Amitraz is hydrolyzed into two main products: *N*-(2,4-Dimethylphenyl)formamide (2,4-DMF) and 2,4-Dimethylaniline (2,4-DMA). The Commission Regulation (EU) No. 37/2010 defines amitraz marker residues as the sum of amitraz and all metabolites containing the 2,4-DMA moiety [8]. According to earlier research by the Environmental Protection Agency (EPA) and the Joint Meeting of Pesticide Residues (JMPR), amitraz toxicity has been linked to genotoxicity, cell death, neurotoxicity, immunotoxicity, endocrine disruption, and other developmental toxicity. However, the dose–response relationship for a number of mechanisms and toxic effects linked to amitraz and its metabolites remain scarcely studied [9]. Therefore, more scientific evidence based on toxicity studies are required. 

On the other hand, the organic compound known as 5-hydroxymethylfurfural (HMF) is a cyclic aldehyde that is considered the most important intermediate product of the acid-catalyzed degradation of hexose as well as the Maillard reaction, a non-enzymatic browning reaction [10]. It is formed when simple sugars such as glucose and fructose, constituents of honey, are degraded due to heat treatment or inadequate storage conditions. As with honey, many foods that contain sugar also include HMF. 

HMF has been shown to have harmful effects on human health in the majority of earlier investigations, including cytotoxicity, mutagenicity and chromosomal abnormalities to both humans and animals [11]. Nonetheless, a wide range of beneficial properties, including antioxidative, anti-allergic and anti-inflammatory, has also been reported for HMF [12]. A daily consumption between 30 and 150 mg of HMF through a variety of dietary items have been estimated; however, safe levels of HMF ingestion are not well clarified since it is still unclear how HMF impacts human health. Hence, the Codex Alimentarius Standard commission established a maximum limit for HMF in honey at 40 mg/kg and at 80 mg/kg for honeys coming from tropical regions [13]. 

Based on previous comments, the objectives of the present study were (i) to determine the cytotoxicity induced by amitraz and its metabolites in human hepatocarcinoma cells (HepG2) and to determine if oxidative stress is one of the mechanisms of action producing cytotoxicity, (ii) to identify and confirm residues of amitraz and its metabolites in samples of honey from amitraz treated hives against *Varroa* infection, and (iii) to evaluate the effect of different heat treatments applied to honey on the stability of amitraz and its metabolites and on the formation of HMF in honey samples.

## 2. Materials and Methods

### 2.1. Reagents

The cell culture compounds as well as the reagent grade chemicals utilized, namely Dulbecco’s Modified Eagle’s Medium (DMEM), penicillin, streptomycin, trypsin/EDTA solutions, Phosphate Buffer Saline (PBS), Newborn Calf Serum (NBCS), methylthiazoltetrazolium salt (MTT) dye, dimethyl sulfoxide (DMSO), sodium hydroxide (NaOH), thiobarbituroc acid (TBA), deferoxamine mesylate salt (DFA), si-ter-butylmethylphenol (BHT), 2′,7′-dichlorodihydrofluorescein diacetate (H2-DCFDA), Coomassie Brilliant Blue, glycine, sodium chloride (NaCl), sodium bisulfite (NaHSO_3_), 4-(2-hydroxyethyl)-1-piperazineethanesulfonic acid (HEPES), calcium chloride (CaCl_2_), formic acid, ammonium formate, magnesium sulfate anhydrous (MgSO_4_), sodium acetate, primary secondary amine (PSA), C18 sorbent, potassium hexacyanoferrate (II) trihydrate (K4[Fe(CN)_6_] × 3H_2_O (Carrez I solution) and zinc sulfate heptahydrate (ZnSO_4 ×_ 7H_2_O) (Carrez II solution), were purchased from Sigma-Aldrich (St Louis, MO, USA). Methanol (MeOH) and acetonitrile (can) were acquired from VWR International (LLC, Radnor, PA, USA). Deionized H_2_O with resistivity <18 MΩ cm was acquired by a Milli-Q water purification system (Millipore, Bedford, MA, USA).

Analytical standards of amitraz, 2,4-DMA and 2,4-DMF were purchased from Sigma-Aldrich (St. Louis, MO, USA). Stock solutions of amitraz and its metabolites were prepared in MeOH at appropriate working concentrations for spiking experiments and stored in the dark at −20 °C.

### 2.2. Cell Culture and Treatment

Human hepatocarcinoma (HepG2) (ATCC: HB-8065) cells were grown in DMEM medium with 10% NBCS, 100 U/mL penicillin and 100 mg/mL streptomycin. The cell line HepG2 were kindly provided by Central Service for Experimental Research (SCSIE) of the University of Valencia (Valencia, Spain). The incubation conditions were pH 7.4, 5% CO_2_ at 37 °C and 95% air atmosphere at constant humidity. Every 5 days, a new medium was used. 

The pesticide concentrations assayed were prepared through the addition of each compound to the medium with a solvent concentration below 1% (*v*/*v*). Controls with the equal concentration of solvents were incorporated in each assay. Absence of mycoplasma was checked by the MycoAlertTM PLUS Mycoplasma Kit (Lonza, Rockland, ME, USA).

### 2.3. In Vitro Cytotoxicity

MTT and total protein content (PC) tests were used in HepG2 cells to analyze cytotoxic effects. They have been widely employed in in vitro toxicological investigations to assess cell survival and proliferation. Only in metabolically active cells can the MTT test detect a cell’s viability by reducing a yellow soluble tetrazolium salt (MTT) to a purple insoluble formazan crystal through a mitochondrial-dependent reaction. According to Ruiz et al. [14], the MTT viability experiment was carried out. On 96-well tissue culture plates, 2 × 10^4^ cells per well were plated.

Based on preliminary laboratory tests, the culture media were replenished after cells reached 80% confluence with a new medium containing serial dilutions of each pesticide (amitraz from 46.88 to 625 µM, and its metabolites 2,4-DMF and 2,4-DMA from 93.75 to 1500 µM). The exposure to pesticides lasted for 24 h. Neither the medium nor the pesticides were replaced during the exposure period. Then, the medium was taken out and 200 μL of fresh medium were introduced into each well. After adding 50 μL/well of MTT solution, the plates were placed back in the incubator in the dark. The MTT solution was removed after 3 h and 200 μL of DMSO were added afterwards followed by the addition of 25 μL Sorensen’s glycine buffer. To achieve the full dissolution, plates were gently shaken for 5 min. The absorbance was determined at 540 nm using an automatic ELISA plate reader (MultiSkanEX, Thermo Scientific, Waltham, MA, USA). 

The PC method is based on the Coomassie Brilliant Blue dye’s increased absorbance when it binds to proteins. The identical 96-well plates used for the MTT test were used for the assay. To dissolve the proteins, 200 μL of NaOH 0.1 N were added to each well after a washing step with PBS and stored in the incubator for 2 h. Then, 170 μL of NaOH were taken out from each well followed by the addition of 180 μL of diluted 22% Coomassie Brilliant Blue. The plates were left for 30 min at room temperature and the absorbance was determined at 620 nm in an automatic ELISA plate reader (MultiSkanEX, Thermo Scientific, MA, USA). 

Cell viability was measured in MTT and PC tests as a percentage in comparison to the control solvent (1% MeOH or DMSO). For each exposure duration, three separate tests were carried out, at least four times for each concentration. Using SigmaPlot version 11 (Systat Software Inc., GmbH, Erkrath, Germany), the mean inhibition concentration (IC_50_) values were calculated.

### 2.4. Determination of Lipid Peroxidation (LPO)

Malondialdehyde (MDA) was measured using the LPO test by reacting with thiobarbituric acid (TBA), which creates an MDA-TBA adduct that is simple to quantify spectrophotometrically. Amitraz, 2,4-DMF and 2,4-DMA concentrations to determine oxidative stress by LPO and intracellular ROS were selected below their IC_50_ values obtained by the two cytotoxicity assays performed in this study. 

The reactive thiobarbituric acid reactive substances (TBARS) method was carried out according to Ferrer et al. [15]. In brief, 5 × 10^5^ cells/well were seeded in 6-well plates. They were exposed to amitraz (78.13, 93.75 and 156.26 µM) and its metabolites, 2,4-DMF and 2,4-DMA (750, 1250 and 1500 µM), for 24 h when the cells reached 80% confluence. Then, the medium was taken out, and the cells were recovered and homogenized in 500 µL of 20 mM Tris + 0.1% triton buffer and lysate with a polytron (Ultra-Turrax T8 IKA-WERKE, GmbH & Co, KG, Staufen, Germany). Afterwards, the cells were exposed to 500 µL of 0.5% TBA, 5 µL of 1.5 mM DFA and 5 µL of 3.75% BHT under acidic conditions and boiled at 100 °C in a water bath for 20 min. Immediately, samples were cooled in ice (5 min), centrifuged (at 252× *g* for 15 min), and the absorbance was determined at 532 nm. Assays were carried out three times and at least in triplicate per concentration. The results were expressed as ng of MDA/mg of protein measured through the Bradford method (mean ± SEM; *n* = 3) [16].

### 2.5. Determination of Reactive Oxygen Species (ROS)

Intracellular ROS accumulation was measured in HepG2 cells by adding H_2_-DCFDA according to Ruiz-Leal and George [17]. The H_2_-DCFDA is taken up by cells and then deacetylated by intracellular esterases; the resulting non-fluorescent 2,7-dichlorodihydrofluorescein (H_2_-DCF) is converted to fluorescent dichlorofluorescein (DCF) when oxidized by ROS. Briefly, 3 × 10^4^ cells/well were seeded in a 96-well black polystyrene culture microplate. After cells reached 80% confluence, the culture medium was replaced and cells were loaded with 20 μM H_2_-DCFDA in a fresh medium for 20 min in darkness. Then, the medium with H_2_-DCFD was removed and the cells were washed with PBS before the addition of a fresh medium (control), control solvent or fresh medium containing different concentrations of amitraz (78.13, 93.75 and 156.26 µM) and its metabolites (750, 1250 and 1500 µM). The increase in fluorescence was monitored in real time (0, 5, 15, 30, 45, 60, 90 and 120 min) with a Wallace Victor^2^ model 1420 (PerkinElmer, Turku, Findland) at excitation and emission wavelengths of 485 and 535 nm, respectively. Measurements were carried out in three independent experiments with 24 replicates each. The results were expressed as an increase (in percentage) of the fluorescence intensity obtained with respect to untreated cells (mean ± SEM; *n* = 3).

### 2.6. Honeybee Colonies Treatment and Sampling

The bee colonies were located in 2020 in two self-beekeeper apiaries in the Camp del Turia region of the Valencian Community (eastern Spain), separated by more than 3 km. Hives were randomly distributed into 2 apiaries with 108 hives in each. The honeybee colonies (*Apis mellifera*) were maintained in Layens hives, equipped with deep bottom boards. During the time of the study, they were treated once a year (autumn for optimum control of the *Varroa destructor*) with the acaricide as an active ingredient (Apitraz ^®^ strips, active substance amitraz 500 mg, Laboratorios Calier, Barcelona, Spain) to avoid the honeybee colonies being infected with V. destructor. In each hive box, there were 12 frames (one-year combs and combs that had been built by bees in the season during the period of study). Two Apitraz strips were hung down between the brood frames in a simple way in the brood nuclei. After 42 days, the strips were removed.

On day 60 after each Apitraz strip was removed, in every colony the hives were distributed in 6 groups of 18 hives. Honey from all the frames of the 18 hives (honeybee colonies) of each group was recollected and pooled in the same containers. Therefore, 6 pooled samples were obtained from each apiary. A total of 12 honey samples were collected (6 samples from each apiary). Fifty grams of honey were transferred to the sterile plastic container and transported and stored separately at 4 °C until analysis.

### 2.7. Sample Preparation and Analysis of Amitraz and Its Metabolites in Honey

The extraction of pesticides from honey samples was carried out as previously reported by Kiljanek et al. [18] with minor modifications. Briefly, 5 g of honey were weighed in a 50 mL propylene tube and dissolved with 10 mL of deionized water and vortexed for 2 min. Then, 10 mL of diluted sample extract were placed into another 50 mL propylene tube and 10 mL of 1% formic acid in acetonitrile were added and vortexed for 2 min. A mixture of salts, which included 4.0 g of MgSO_4_ and 1 g of sodium acetate, was added to the tube and was vortexed again for 2 min and centrifuged at 4 °C and 252× *g* for 3 min (X3R Heraeus Multifuge, Thermo Fisher Scientific, Madrid, Spain). After centrifugation, the supernatant was collected and transferred into a 15 mL propylene tube in which 350 mg of C18 sorbent, 350 mg of PSA, and 1.0 g of MgSO_4_ were weighed. The mixture was vortexed for 1 min and centrifuged at 4 °C and 252× *g* for 3 min. The upper layer was collected into another 15 mL propylene tube and evaporated to dryness under a gentle N_2_ stream at 45 °C with a TurboVap-LV (Zymark, Allschwil, Switzerland). The residue was reconstituted with 250 μL of MeOH:H_2_O (70:30, *v*/*v*) and then filtered through a 0.2 μm PTFE filter prior to the analysis.

The analysis was carried out using an Agilent 1200 Infinity Series LC system (Agilent Technologies, Santa Clara, CA, USA) coupled to an Agilent Technologies 6540 UHD Accurate-Mass Q-TOF (Agilent Technologies, Santa Clara, United States) equipped with an Agilent Technologies Dual Jet Stream electrospray ionization (Dual AJS ESI) (Agilent Technologies, Santa Clara, United States). Chromatographic separation was carried out by a Gemini-NX column C18 (110 Å, 3 μm, 150 mm × 4.6 mm) (Phenomenex; Torrance, CA, USA) with the temperature set at 20 °C. The chromatographic gradient consisted of water (phase A) and MeOH (phase B), both containing 0.1% formic acid and 5 mM ammonium formate. The elution gradient was: 50% phase B maintained for 6 min, followed by a linear ascent to 100% phase B in 1 min and held for 5 min. The gradient decreased to 50% phase B in 1 min and sustained for 7 min to allow for column re-equilibration. Total run time was 20 min with a flow rate set at 200 µL/min and an injection volume of 5 µL.

The mass spectrometry (MS) analysis was carried out throughout the following settings: drying gas temperature, 330 °C; drying gas flow (N_2_), 10 L/min; sheath gas flow, 9 L/min; sheath gas temperature, 350 °C; and nebulizer pressure, 30 psi. The Q-TOF mass spectrometer operated in AutoMS/MS in positive ionization mode. The ion source parameters were: nozzle voltage, 500 V; capillary voltage, 3500 V; skimmer voltage, 30 V; octopole RF peak, 750 V; and fragmentor voltage, 160 V. Internal mass correction was carried out through two reference masses: 121.0509 and 922.0098 *m*/*z*. 

Instrument control and data acquisition were performed using Agilent MassHunter Workstation software B.08.00 (Agilent Technologies, Santa Clara, CA, USA). The identification of amitraz and its metabolites was verified by comparing the retention times of peaks in positive samples to those shown by analytical standards, setting a strict mass accuracy of 5 ppm in relation to their corresponding theoretical mass. As a result, the HPLC-HRMS combination supplied the three identification points needed to comply with Commission Decision 2002/657/EC [18] for confirmation criteria. Table 1 shows the HPLC-HRMS parameters of amitraz and its metabolites.

### 2.8. Method Validation

According to the EU Commission Decision 2002/657/EC standards related to linearity, selectivity, trueness, and sensitivity expressed as limits of quantification (LOQs), an internal validation study was carried out [19]. At eight concentration ranges ranging from LOQ to 250 ng/g, linearity was assessed across plain solvent and matrix-matched calibration curves. The least squares method was used to obtain the coefficient of determination. The slopes corresponding to the calibration curve built in neat solvent (A) and blank matrix (B) were compared to evaluate matrix effects. As a result, the ratio (B/A × 100) may show an enhancement (ratio > 100%) or suppression (ratio 100%) that might affect the quantitative findings. By injecting blank samples (*n* = 20), looking for any peaks that coeluted within the same retention time region as the analytes, and always accounting for a mass error of 5 ppm, the method’s selectivity was evaluated. Trueness was assessed using recovery tests that spiked three blank samples at three different fortification levels corresponding to amitraz maximum residue limit (MRL), MRL/2 and MRL/10. The measurements were taken over the course of three separate days. Relative standard deviation after three measurements in a single day (RSD_r_) and relative standard deviation after three non-consecutive days of determinations in triplicate (RSD_R_) were used to assess the precision. The LOQ for each analyte, which was set as the minimal concentration inside the linear range that could be seen with a variation of 20% and taking into account a mass error of 5 ppm, was used to assess sensitivity. 

Data quality was verified through the inclusion of quality assurance and quality control procedures. For confirmation criteria, the retention times of analytes in standards and samples were compared (tolerance of ±2.5%). Each batch of samples included a procedural blank, a reagent blank and a matrix-matched calibration for the evaluation of the robustness and stability through the analysis.

### 2.9. Determination of 5-hydroxymethylfurfural (HMF)

The HMF concentration in the honeybee samples was measured according to the official AOAC method [20]. Briefly, 5 g of honey were dissolved in 25 mL of deionized water and transferred to a 50 mL volumetric flask. Then, 0.5 mL of Carrez solution I and 0.5 mL of Carrez solution II were added and made up to 50 mL with water. The solution was filtered through paper rejecting the first 10 mL of filtrate and then collecting two aliquots of 5 mL, which were placed into two test tubes. Afterwards, 5 mL of deionized water were added to one tube (sample solution) and 5 mL of NaHSO_3_ 0.2% were added to the second tube (reference solution). The absorbance of the solutions was measured at 284 and 336 nm by using a Shimadzu^TM^ UVmini-1240 model spectrophotometer (Fisher Scientific, Dublin, Ireland). All determinations were carried out in triplicate and the results were expressed as mean ± SD (mg/kg).

### 2.10. Thermal Stability of Amitraz and Its Metabolites

The thermal stability of amitraz and its metabolites, as well as the formation of HMF through non-enzymatic browning reactions, were evaluated to determine the effect of different heat treatments that are applied on an industrial scale on honey to improve its physicochemical characteristics. In detail, 25 g of honey samples containing amitraz at different levels (previously confirmed by the HPLC-HRMS methodology) were introduced into a scalder and subjected to the following treatments: (a) 50 °C for 2 min, (b) 65 °C for 25 min, and (c) 75 °C for 2 min. 

Once the honey samples were tempered, the procedure described in points 2.7 and 2.9 was followed to determine the values of amitraz and its metabolites and HMF, respectively.

### 2.11. Statistical Analysis

Using the statistical tool SPSS version 24.0, the data were statistically analyzed (IBM Corp., Armonk, NY, USA). Standard error of the mean (SEM) was used to express data from several separate experiments. The Student *t*-test for paired samples was used for the statistical analysis of the findings. One-way analysis of variance (ANOVA) and the Tukey HDS post hoc test for multiple comparisons were used to examine group differences. Statistical significance was considered for *p* ≤ 0.05.

## 3. Results

### 3.1. Oxidative Stress Induced by Amitraz and Its Metabolites

**Figure 1:**
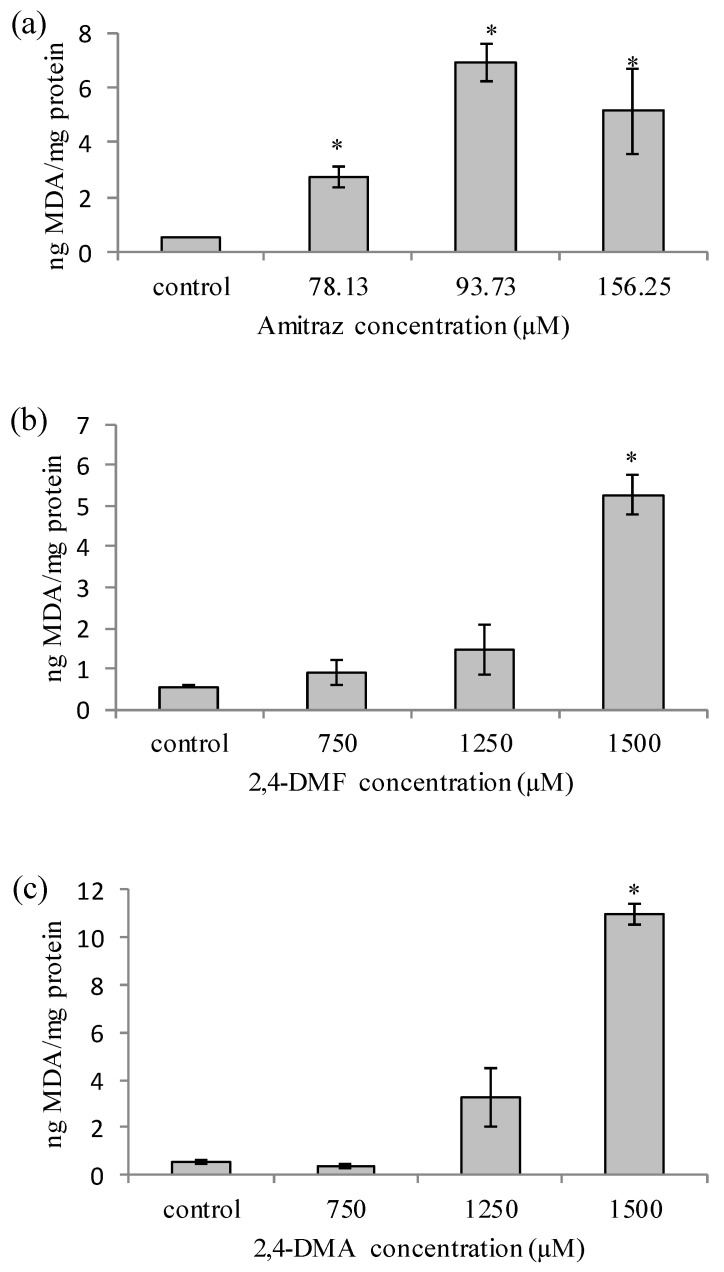
Lipid peroxidation production in HepG2 cells for (**a**) amitraz, (**b**). *N*-(2,4-Dimethylphenyl)formamide (2,4-DMF) and (**c**) 2,4-Dimethylaniline (2,4-DMA) after 24 h of exposure. Results are expressed as mean ± SEM (*n* = 3) in ng of Malondialdehyde (MDA)/mg of protein measured by Bradford method. (*) *p* ≤ 0.05 indicates significant differences from control.

To understand the role of amitraz and its metabolites on the oxidative stress, the LPO has been evaluated by the TBARS method in HepG2 cells at 24 h of exposure. The results showed that amitraz significantly increased the LPO (Figure 1a) at all the concentrations tested in HepG2 cells. Additionally, 2,4-DMF and 2,4-DMA only increased the LPO in HepG2 cells at the highest concentration tested (1500 µM) (Figure 1b,c).

**Figure 2:**
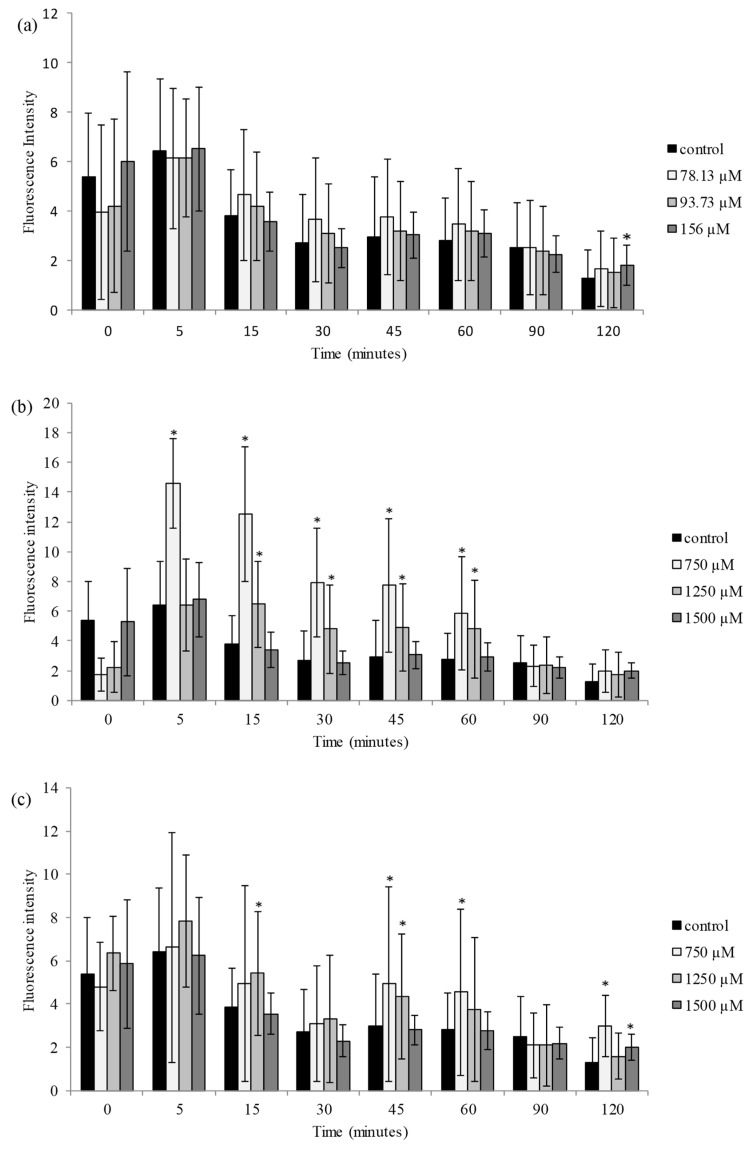
Time dependence of ROS-induced fluorescence in HepG2 cells for (**a**) amitraz, (**b**) 2,4-DMF and (**c**) 2,4-DMA. Fluorescence of the oxidized diclhorofluoresceine was followed by emission and excitation wavelengths at 535 and 485 nm, respectively. Results are expressed as mean ± SEM (*n* = 3). (*) *p* ≤ 0.05 indicates significant differences from control.

To evaluate the role of ROS production in pesticide-mediated cytotoxic effects in HepG2 cells, the production of the fluorescent DCF in response to different concentrations of amitraz and its two metabolites was analyzed. As observed in Figure 2a, no significant increase of ROS production was measured in HepG2 cells at the three concentrations of amitraz tested in the interval 0–90 min. However, a significant slight increase in ROS generation (1.02 folds of control) was observed at the highest concentration (156.25 µM) tested at 120 min with respect to the control. Regarding 2,4-DMF, the results showed an increase in ROS production at 750 and 1250 µM from 5 to 60 min (Figure 2b). The increase in ROS production ranged from 2.11- to 3.27-fold and from 1.66- to 1.77-fold with respect to control in HepG2 cells, respectively. As shown in Figure 2c, ROS generation after 2,4-DMA exposure was significant at the three concentrations tested at different time ranges. The highest fluorescence intensity (2.3-fold with respect to control) was observed at 750 µM after 120 min.

### 3.2. Analytical Characteristics of the Proposed Method

The results of the in-house validation method for the determination of amitraz, 2,4-DMF and 2,4-DMA in honey samples are shown in Table 2. Correlation coefficients (r) above 0.994 were obtained within the evaluated linear range. The matrix effects were in the range from 83% to 94%, resulting in negligible interference of the matrix and therefore neat solvent calibration curves were used for quantification purposes. 

**Figure 3:**
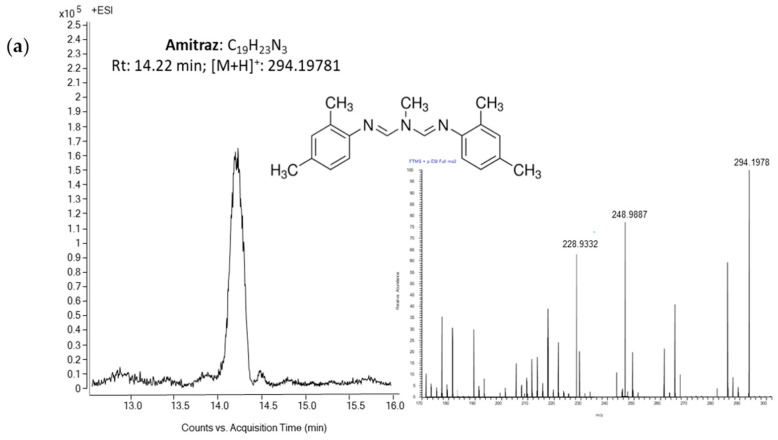

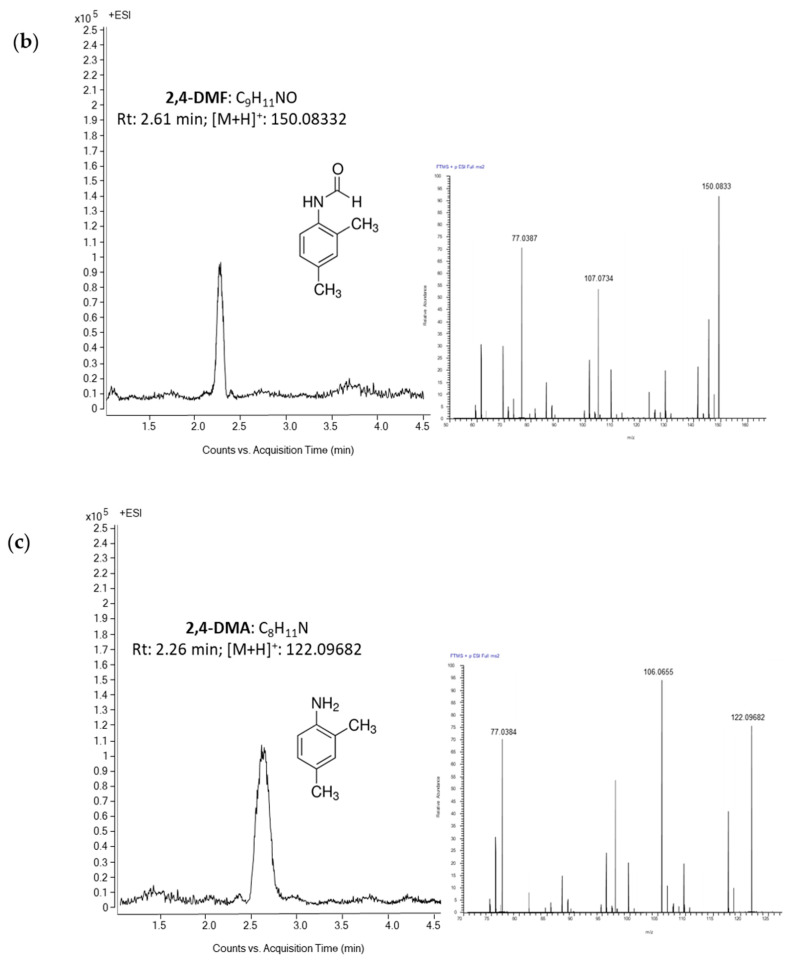
High-performance liquid chromatography-high resolution mass spectrometry (HPLC-HRMS) chromatograms of a honey sample spiked with (**a**) amitraz, (**b**) 2,4-DMF and (**c**) 2,4-DMA at 20 ng/g (corresponding to amitraz MRL/10).

An appropriate selectivity was achieved observing no co-elutant peaks within the elution time of the investigated pesticides after the injection of blank samples. High sensitivity was also displayed by the applied methodology, enabling the quantification of analytes under study at LOQs within a range 0.05–0.1 ng/g. Trueness was assessed through spiking experiments at levels of MRL, MRL/2 and MRL/10 and recovery values ranged between 78% and 93%, and the method was repeatable (RSD_r_ < 11%) and reproducible (RSD_R_ < 18%). Figure 3 shows a HPLC-HRMS chromatogram of a honey sample spiked with amitraz, 2,4-DMF and 2,4-DMA at 20 ng/g (corresponding to amitraz MRL/10).

### 3.3. Determination of Pesticides in Honey Samples from Amitraz-Treated Honeybee Colonies

To demonstrate the suitability of the validated method, it was applied to the analysis of 12 honey samples. The results are shown in Table 3. Amitraz or its metabolites were detected in nine out of the twelve honey samples from amitraz-treated honeybee colonies with this pesticide to control *Varroa* mites in hives. In all cases, the quantified concentration of 2,4-DMA, its main metabolite, was higher than the parent compound. The range of concentrations of amitraz and its metabolites quantified in the tested samples was from 6.8 to 181.5 ng/g. However, all positive samples comply with the MRL set in the current legislation.

### 3.4. Determination of 5-hydroxymethylfurfural (HMF) in Honey Samples

The HMF content of the analyzed honey samples are shown in Table 3. HMF was quantified in all samples ranging between 2.2 and 17.4 mg/kg. The observed concentrations of this product of non-enzymatic browning reactions are within the limits contemplated in the legislation. However, it should be noted that in two out of the twelve honey samples directly collected from the hive, values higher than 10 mg/kg of HMF were detected, with these values being more typical of older honey.

### 3.5. Effect of Heat Treatments on Amitraz Stability and HMF Formation 

In addition to the analysis of HMF in the collected samples (room temperature, *n* = 12), a parallel study was carried out in three amitraz positive samples with different levels of amitraz (sample number 5: 40.7 ng/g; sample number 10: 181.5 ng/g and sample number 12: 104.7 ng/g, see Table 3) to determine the effect of different heat treatments that are applied on an industrial scale on honey to improve its physicochemical characteristics on: (i) the stability of amitraz and its metabolites, and (ii) the formation of HMF through non-enzymatic browning reactions. The results confirmed that the heat treatment to which honey is subjected (regardless of the combination of temperature and time tested, see Section 2.10) were sufficient to degrade amitraz and its metabolites, not detecting the presence of any of them in the assayed samples. The values of amitraz and its metabolites, reported in Table 3 (no heat treatment applied to samples), serve as control values. As far as the authors’ knowledge, there is no report on the kinetic evaluation of amitraz residues in honey during thermal treatments. Based on the findings, it can be highlighted that temperature plays an important role in the degradation of amitraz. This fact should be further studied because it is important for the reduction of dietary exposure to the residues, which pose a serious threat to human health.

On the other hand, the results obtained showed a positive correlation in terms of HMF concentration in the samples and severity of the heat treatments. With the most moderate treatment (50 °C for 5 min), an HMF range between 0.8 and 7.5 mg/kg was obtained (average content: 4.9 ± 2.5, *n* = 9). However, the most severe heat treatments showed a significantly higher HMF level. For 75 °C and 2 min, an interval of HMF from 3.9 to 39.7 mg/kg was obtained (average content: 28.9 ± 10.4, *n* = 9), and for the longest heat treatment (65 °C for 25 min) an HMF interval between 4.2 and 45.6 mg/kg was obtained (average content: 34.8 ± 12.7, *n* = 9).

## 4. Discussion

The harmful effects of amitraz and its metabolites, 2,4-DMF and 2,4-DMA, were examined in the current study. In addition, researchers looked at potential mechanisms of oxidative stress brought on by pesticides in HepG2 cells. It is widely known that amitraz can cause significant dysfunctions in both people and animals after an acute exposure [21,22]. As the liver is the first organ where the toxins are metabolized, the HepG2 cells have been chosen as the experimental system in vitro to detect toxicity generated by amitraz, 2,4-DMF, and 2,4-DMA. The HepG2 cells have a large number of mitochondria organelles and nevertheless exhibit the activity of several phase I and II enzymes [23]. HepG2 cells have been found to express less cytochrome P450 than the human liver, which results in less biotransformation activity [24]. In comparison to other widely used cell lines, such as HeLa or Caco2 cells, the HepG2 cells were also demonstrated to be somewhat more susceptible to cytotoxic substances.

For cell culture, a variety of in vitro cytotoxicity tests with various outcomes are available. The cytotoxic effect of amitraz (from 46.88 to 625 µM) and its metabolites 2,4-DMF and 2,4-DMA (from 93.75 to 1500 µM) was evaluated by the MTT assay and PC assay after 24 h of exposure in a previous study carried out in our laboratory [9]. Amitraz significantly decreased cell viability from 31% to 100% at 156.25 μM by MTT assay and from 69% to 100% at 312.5 μM by PC assay. No significant reduction in cell proliferation was produced by 2,4-DMF in HepG2 cells after 24 h of exposure in any of the two assays. However, the 2,4-DMA decreased cell proliferation at 1500 µM by MTT (26%) and PC (53%) assays after 24 h of exposure. Amitraz was the only pesticide that showed IC_50_ values in HepG2 cells by MTT (196.5 ± 10.1) and PC (260.0 ± 13.3) assays. The 2,4-DMF and 2,4-DMA did not show any IC_50_ values within the range of concentrations tested, being less cytotoxic than amitraz.

We may draw the conclusion that both the MTT and PC tests were sensitive and similar for assessing the cytotoxicity caused by amitraz and its metabolites. Nevertheless, little research has been carried out to establish the toxicity of amitraz and its metabolites in cell cultures. Using the MTT test for 24 h, the cytotoxic impact of amitraz (from 60 to 120 M) on primary hippocampus cells was assessed. Up to 100 µM, Amitraz reduced the viability of the cells in a concentration-dependent way [25]. Similar results were obtained by del Pino et al., who used the MTT test to track the metabolic activity of primary hippocampal cells for 24 h after exposure to 100 µM amitraz [26]. Young et al. [27] have shown the cytotoxicity of amitraz (from 0.119 to 119 µM) in human lymphoblastoid (WIL2NS) cell lines after 24, 48, and 72 h. With the exception of the lowest measured concentration, they noticed a substantial decline in cell viability for all concentrations examined.

Oxidative stress defines the outcome of an imbalance between pro-oxidant (ROS) and antioxidant molecules, which results in a wide range cell damages. The overproduction of ROS may induce cell oxidative injury, such as DNA damage, oxidation of proteins and LPO [28]. To determine if cytotoxicity produced by amitraz, 2,4-DMF and 2,4-DMA is related to the oxidative stress, LPO generation and intracellular ROS production were evaluated in HepG2 cells. The results of this study showed a significant increase in MDA production after 24 h of amitraz exposure at all concentrations tested. However, an increase in MDA production after 2,4-DMF and 2,4-DMA exposure was observed after 1500 µM. In the literature, data about LPO production by amitraz and its metabolites in cell cultures are scarce. According to Moyano et al. [25], both amitraz and one of its mainly aminobenzotiazole metabolites (BTS-27271), which are lipophilic compounds, cross cell membranes and enhance LPO. They detected a significant increase in MDA levels in primary hippocampal cell exposed 24 h to 100–120 µM of amitraz and 80 µM of BTS-27271 compared to control cells. Until now, no data about LPO generation by amitraz and its metabolites have been published on cell lines other than hippocampal cells. Hence, we decided to explore if these pesticides could increase the early generation of ROS in HepG2 cells. Our results demonstrated that there was a significant increase in ROS generation after 120 min of amitraz exposure only at the highest concentration (156.25 µM) tested. However, a significant increase in ROS generation was observed at lower 2,4-DMF and 2,4-DMA concentrations (Figure 2). Similarly to our results, several authors have reported that amitraz increased ROS levels at 80 µM in a concentration dependent manner after 24 h exposure in primary hippocampal neurons [25]. Therefore, the results obtained by these authors support our findings.

As far as pesticides determination is concerned, we observed that amitraz rapidly degrades itself to its final metabolite 2,4-DMA, which is the main amitraz metabolite found in honey samples (Table 3). However, despite the fact that the honeybee colonies were treated by acaricidal strips with amitraz and these colonies produced honey that was sampled, none of them exceeded the levels of amitraz (total sum) established in the legislation, that is 0.2 mg/kg. 

Other authors have analyzed amitraz and its metabolites in honey and beehive products. Table 4 shows that the reported concentrations of those pesticides in the literature are in the same concentration range as those obtained in our study. Kubiak and Biesaga [29] analyzed amitraz and its metabolites in honey samples stored for longer than 4 weeks, and they observed that only the final degradation products of amitraz (DMF and 2,4-DMA) were detected and at levels below the permitted MRLs. 

Li and coworkers [30] collected 35 honey samples to determine the amitraz and its metabolites. They found five positive samples with amitraz or 2,4-DMA residues (Table 4). The highest detected amount of amitraz was 11 µg/kg. Zheng et al. [31] analyzed a total of 43 honey and royal jelly samples to determine the amitraz and 2,4-DMA concentrations. Amitraz was not detected in any of the samples, whereas DMF and 2,4-DMA were detected in 37 samples, but not quantified. 

Pohorecka and coworkers [32] investigated two different methods of amitraz application in hives. The hives were fumigated four times every four days with one tablet of 12.5 mg amitraz or given at the same time, but with indirect smoke generation (tablets of 12.5 mg amitraz were combusted in a Wakont electric fumigator) and introduced to entrances of hives through a nozzle. The results obtained by Pohorecka et al. [33] demonstrated that a higher concentration of amitraz residues in honey was observed with the application of amitraz directly inside the beehive. Moreover, they observed a significant impact on the concentrations of amitraz residues when treatment was repeated several times. Five honey samples exceeded the MRL established for amitraz after the four applications by both methods. 

It has to be remarkable that the quality of honey is impacted by industrial processing of commercial honeys during extraction and storage. Thermal processing, which is one of the most used procedures in the honey business, may alter the physicochemical characteristics of honey. In this line, several authors demonstrated that amitraz was nearly completely degraded within 10 days in honey even at room temperature [33]. However, it is important to consider that high temperatures increase the HMF levels in honey, which could overcome the set limit by regulation as evidenced in the literature [34]. The results obtained in this study demonstrated that the different heat treatments tested completely degraded the amitraz and its metabolites. Additionally, the highest HMF content in honey samples was obtained in those samples subjected to more severe and prolonged heat treatments. Similar findings were also reported by Bodor et al. [34].

**Table 4:** Concentrations of amitraz and its metabolites in honey and other bee-products.

Matrix	Number of Samples	Positive Samples (%)	Amitraz (ng/g)	2,4-DMF (ng/g)	2,4-DMA (ng/g)	Reference
Honey	43	86.0	n.d.	<LOQ	<LOQ	[31]
Honey	54	96.1	n.d.	1.0–29.9	10.0–309.5	[32]
Honey	5	n.d.	<LOQ—11.0	<LOQ—420	<LOQ—180	[29]
Honey	35	14.3	<LOQ—11.0	n.d.	<LOQ—4.65	[30]
Honey	16	6.2	n.d.	<LOD—60.5	n.d.	[35]
Beeswax	16	18.8	n.d.	<LOD—6160	<LOD—177	[35]

n.d.: not detected; LOD: limit of detection; LOQ: limit of quantification.

## 5. Conclusions

In summary, our study demonstrated that the pesticide amitraz was more cytotoxic than its metabolites 2,4-DMA and 2,4-DMF. These three pesticides caused oxidative stress through LPO production and ROS generation. Amitraz and its metabolites have been detected in several honey samples, but the concentrations found were lower than the set limit. Heat treatment to honey degraded the studied pesticides, but increased the HMF content in samples. Therefore, as a general conclusion according to the results obtained in this study, it is possible to assume that the honey samples analyzed here are safe for the consumer. 

## Figures and Tables

**Table 1:** High-performance liquid chromatography-high resolution mass spectrometry (HPLC-HRMS) parameters of amitraz, *N*-(2,4-Dimethylphenyl)formamide (2,4-DMF) and 2,4-Dimethylaniline (2,4-DMA) used for their determination in honeybee samples.

Pesticide	RT (min)	Elemental Composition	Adduct Ion	Theoretical Mass (*m*/*z*)	Measured Mass (*m*/*z*)	Mass Accuracy (∆ mg kg^−1^)
Amitraz	14.22	C_19_H_23_N_3_	[M+H]^+^	294.19647	294.19781	4.55
2,4-DMF	2.61	C_9_H_11_NO	[M+H]^+^	150.08406	150.08332	−4.93
2,4-DMA	2.26	C_8_H_11_N	[M+H]^+^	122.09697	122.09682	−1.23

RT: retention time.

**Table 2:** The validation parameters of the optimized method for amitraz, 2-4-DMA and 2,4-DMF in honey.

Pesticide	Linearity (r2)	LOQ (ng/g)	Recovery (%)			Intra-Day Precision (RSD_r_, %)			Inter-Day Precision (RSD_R_, %)		
			MRL/10	MRL/2	MRL	MRL/10	MRL/2	MRL	MRL/10	MRL/2	MRL
Amitraz	0.9965	0.10	84	87	93	9	9	5	18	15	12
2,4-DMF	0.9940	0.05	78	85	91	11	9	4	16	13	11
2,4-DMA	0.9956	0.05	91	92	88	8	7	6	16	9	14

LOQ: limit of quantification; MRL: maximum residue limit of amitraz (0.2 mg/kg); RSD_r_: relative standard deviation after three measurements in a single day; RSD_R_: relative standard deviation after three non-consecutive days of determinations in triplicate.

**Table 3:** Determination of pesticides (amitraz, 2,4-DMF and 2,4-DMA) and hydroxymethylfurfural (HMF) in analyzed honey samples.

Sample	Amitraz (ng/g)	2,4-DMF (ng/g)	2,4-DMA (ng/g)	Total Amitraz (Sum of Amitraz and Its Metabolites) (ng/g)	HMF (mg/kg)
1	<LOQ	<LOQ	<LOQ	-	2.5
2	<LOQ	<LOQ	<LOQ	-	6.6
3	<LOQ	<LOQ	<LOQ	-	2.2
4	10.2	<LOQ	51.6	61.8	4.5
5	8.6	<LOQ	32.1	40.7	3.7
6	<LOQ	<LOQ	6.8	6.8	4.9
7	50.6	<LOQ	79.2	129.8	7.8
8	25.7	<LOQ	66.7	92.4	6.3
9	<LOQ	<LOQ	12.4	12.4	8.5
10	78.3	<LOQ	103.2	181.5	17.4
11	<LOQ	<LOQ	35.9	35.9	10.6
12	29.6	<LOQ	75.1	104.7	2.4

LOQ: limit of quantification.

## Data Availability

Data are contained within the article.
